# Novel extraction technique of retained pacemaker and defibrillator lead during heart transplantation

**DOI:** 10.1371/journal.pone.0203172

**Published:** 2018-09-06

**Authors:** Eriko Hasumi, Katsuhito Fujiu, Toshiya Kojima, Osamu Kinoshita, Kan Nawata, Haruo Yamauchi, Minoru Ono, Issei Komuro

**Affiliations:** 1 Department of Ubiquitous Health Informatics, Graduate School of Medicine, The University of Tokyo, Tokyo, Japan; 2 Department of Cardiovascular Medicine, Graduate School of Medicine, The University of Tokyo, Tokyo, Japan; 3 Department of Advanced Cardiology, Graduate School of Medicine, The University of Tokyo, Tokyo, Japan; 4 Department of Cardiac Surgery, Graduate School of Medicine, The University of Tokyo, Tokyo, Japan; Nagoya University, JAPAN

## Abstract

**Background:**

Removal of cardiac implantable electronic devices (CIEDs) by manual traction during orthotopic heart transplantation (OHT) sometimes results in retained lead fragments. Moreover, abandoned leads and retained lead fragments are a contraindication for magnetic resonance imaging (MRI) and may be a cause of CIED infection.

**Objective:**

To eliminate complications of retained lead fragments, we completely removed residual leads using an excimer laser sheath technique during OHT. We report our clinical experience and high success rate of lead extraction using the excimer laser sheath compared with manual traction during OHT.

**Methods and results:**

We obtained data on 84 consecutive patients receiving OHT between August 2007 and August 2017. Thirty-nine of 84 patients had undergone CIED implantation before OHT and removal of all their leads was attempted during OHT. From 2007 to 2014, defibrillator and pacemaker leads were extracted by manual traction in all patients (N = 22). After 2015, all leads were extracted with the excimer laser sheath, and surgical assistance was prepared for the procedure (N = 17). Complete procedural success was achieved in 100% of patients in the excimer laser group and 77% of patients in the manual traction group.

**Conclusion:**

Extraction of abandoned leads using the excimer laser sheath system during OHT is novel and safe technique, and has a higher success rate than extraction using manual traction during OHT.

## Introduction

Implantations of an implantable cardioverter defibrillator (ICD) and a cardiac resynchronization therapy defibrillator (CRTD) have been demonstrated to reduce sudden death and improve total mortality in patients awaiting orthotopic heart transplantation (OHT) [1]. Although surgical operators have attempted to remove abandoned leads originating from these devices using manual tractions during OHT, many cases (19 to 39% of patients after OHT) experienced retention of leads from devices that had been implanted for a long period [2, 3]. Although, long-term clinical outcomes of retained leads remain unclear, one cohort study reported short-term outcomes of retained transvenous leads after OHT [3]. According to this report, the presence of retained leads was not associated with mortality rate within an observed period. However, they also reported cases of residual lead erosion into the mediastinum and embolism in the pulmonary artery [3]. Moreover, two other cases have been reported to have retained lead fragments that induced asymptomatic pulmonary embolism and perforation of the left ventricle [4]. To prevent these complications and future potential hazards relating to retained leads, we attempted to perform a complete extraction of implanted leads using the excimer laser sheath system during OHT. To date, there are no preceding reports demonstrating the extraction of leads using the excimer laser sheath method during OHT. Therefore, we report a novel and safe lead extraction technique which had a higher success rate of residual lead extraction using excimer laser sheath than that using conventional manual lead traction during OHT.

## Method

### Study participants and data collection

**Fig 1:**
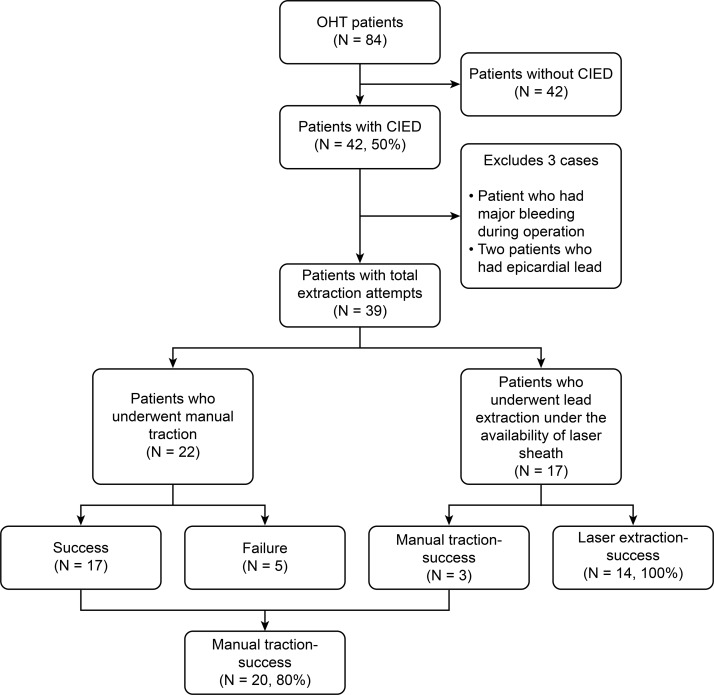
Summary of this study. This study is a historical cohort study. Before 2015, manual extractions were performed during OHT. After 2015, the excimer laser sheath extraction system was prepared for use during OHT. After 2015, manual extraction was attempted at first, and if the leads tightly adhered to the vessel wall, excimer laser sheath was used.

We retrospectively collected data from 84 consecutive patients undergoing OHT at the University of Tokyo Hospital between August 2007 and August 2017 Forty-two of 84 (50%) patients were implanted with cardiac implantable electronic devices (CIEDs), and 39 patients, excluding 3 patients (2 patients who had epicardial leads and one patient whose leads not extracted due to bleeding), underwent transvenous lead extraction during OHT. In 22 patients, implanted leads were extracted by manual traction during OHT from 2007 to 2014. In 17 patients, implanted leads were extracted under availability of excimer laser sheath from 2015 to present (Fig 1). This historical cohort study was approved by the institutional ethical committee of the University of Tokyo (No. 2650–6), and written informed consent was obtained from all participants of this study.

### Retained lead removal during OHT by excimer laser sheath

When a recipient heart was excised, intracardiac portions of lead were cut off at the superior vena cava using surgical scissors, and a donor heart was anastomosed to the recipient vessels. After weaning from cardiopulmonary bypass, heparin was reversed with protamine and activated cloting time (ACT) of <150 seconds was targeted as a timing of lead extraction. Removal of residual leads from the superior vena cava and subclavian vein was attempted during open-chest surgery. First, the device pocket was opened, the CIED generator was exposed and the leads were surgically explored up the entry side into the subclavian or axillary vein.

**Fig 2:**
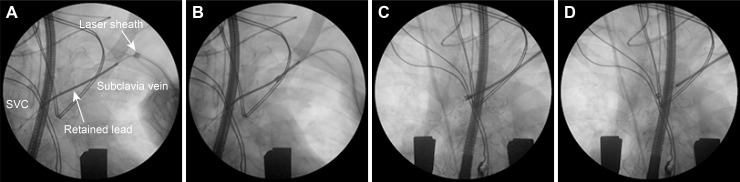
Retained lead extraction using the excimer laser in a hybrid operating room. (A) Insertion and One-Tie fixation of a locking stylet into retained lead. B-D) Excimer laser sheath advancing over the lead with laser ablation and complete extraction of retained lead.

From 2007 to 2014, all residual leads were manually extracted during OHT. The operators pulled to the extent that injury of blood vessels or breaking lead did not occur. From 2015, the procedure for lead removal during OHT involved two steps. First, we tested manual traction and evaluated whether residual leads could be easily extracted manually. If residual leads could not be easily extracted, lead extractions using the excimer laser sheath were performed as the second method. In case of manual traction, leads were removed by simple traction without fluoroscopic guidance. In cases of extraction using excimer laser sheath, an appropriately sized lead locking device (LLD) locking stylet (Spectranetics, USA or Liberator^Ⓡ^; Cook MEDICAL, USA) was advanced towards the distal end of the cut along the inner lumen and deployed. Surgical sutures or a One-Tie Compression Coil^Ⓡ^ (Cook MEDICAL, USA) were used to securely bind proximal components of the cardiac lead to the inserted locking stylet. SLS II^Ⓡ^ sheaths (Spectranetics, USA) that were appropriately size-matched for the diameter of the residual lead were advanced over the lead, and the adhesion tissue between the lead and blood vessel wall was ablated using a CVX-300 Excimer Laser System (Spectranetics, USA) (Fig 2). After lead extraction, the device pocket was closed by sutures.

### Definitions

Complete success of the procedure was defined as the removal of all targeted leads and all lead material from the vascular space without the occurrence of any permanently disabling complication or procedure-related death. Complications were defined as outcomes that were life-threatening, resulted in significant or permanent disability or death, or required surgical or medical intervention.

### Statistical analyses

We compared baseline demographics, implanted devices, and the occurrence of cardiovascular disease between patients. Statistical significance was tested between the groups using a chi-square test for categorical variables. All analyses were two-sided, and a *P* value less than 0.05 was considered statistically significant. All statistical analyses were performed using SPSS software, version 23.0 (SPSS Inc., Chicago, IL).

## Results

Of the 84 patients who underwent OHT during the observation period, 42 patients (50%) received a device implantation: 31 patients (74%), 4 patients (9.5%), 4 patients (9.5%), 1 patient (2.4%), and 2 patients (4.8%) received CRTD, ICD, cardiac resynchronization therapy pacemaker (CRTP), pacemaker (PM), and epicardial lead alone, respectively. The mean implantation period was 1775±139 days. Thirty-nine of the 42 patients underwent transvenous device extraction (Table 1). Lead removal was conducted by manual traction in 25 patients, and the lead was removed using a laser sheath in 14 patients (Table 2). In the manual traction group, retained leads were observed in 5 patients (Fig 1, Table 1), but there was no significant difference in the outcomes between the patients with retained leads after manual extraction and the patients without retained leads (Table 1). However, the implantation period was 2331±490 days and 1765±148 days in the group with retained leads and group without retained leads, respectively, which showed a tendency that the implantation period was slightly longer in the group with retained leads (*P* = 0.11) (Table 1).

**Table 1:** Clinical characteristics of the study group.

		overall(N = 84)	with retained lead after extraction (N = 5)	without retained lead after extraction(N = 34)	*P* value
age	* *	38.6±13.9	40.0±11.4	41.6±11.0	0.39
male		60 (71%)	2 (40%)	26 (76%)	0.25
etiology	ICM	10 (2.5%)	0 (0%)	1 (2.9%)	0.7
	DCM	61 (73%)	4 (80%)	26 (76%)	0.86
	HCM	5 (6.0%)	0 (0%)	4 (12%)	0.98
	others	8 (9.5%)	1 (20%)	3 (8.8%)	0.44
implanted CIED or lead		42 (50%)			
type of device	ICD	4 (9.5%)	2 (40%)	2 (11%)	0.12
	CRTD	31 (74%)	2 (40%)	28 (72%)	0.13
	CRTP	4 (9.5%)	1 (20%)	3 (11%)	0.44
	PM	1 (2.4%)	0 (0%)	1 (%)	0.7
	none*	2 (4.8%)			
median time from device implantation to OHT (days)		1775±139	2331±490	1765±148	0.11
type of defibrillator lead	single coil		1(20%)	3 (13%)	0.44
	dual coil		4(80%)	27 (87%)	0.98
number of leads at time of OHT	1		1(20%)	1 (2.9%)	
	2		2(40%)	4 (12%)	
	3		2(20%)	27 (79%)	
	4		0(0%)	1 (2.9%)	
	5		0(0%)	1 (2.9%)	
average number of implanted leads			2.4±0.9	2.9±0.7	0.16

ICM, ischemic cardiomyopathy; DCM, dilated cardiomyopathy; HCMs, hypertrophic cardiomyopathy; CIEDs, cardiac implantable electronic devices; ICD, implantable cardioverter defibrillator; CRTD, cardiac resynchronization therapy defibrillator; CRTP, cardiac resynchronization therapy pacemaker; PM, pacemaker; OHT, orthotopic heart transplantation; *P* value shows with vs without retained lead.

*epicardial lead without device

**Table 2:** Background in patients with retained leads and patients without retained leads using the excimer laser sheath.

		with retained leads after manual traction (N = 5)	excimer laser sheath extraction(N = 14)	P value
type of device	ICD/CRTD	4 (80%)	14 (100%)	0.09
	CRTP	1 (20%)	0 (0%)	0.09
median time from device implantation to OHT (days)		2331±490	2099±219	0.34
type of defibrillator lead	single coil	1 (20%)	3 (21%)	0.95
	dual coil	4 (80%)	11 (79%)	0.95
number of leads at time of OHT	1	1 (20%)	0 (0%)	
	2	2 (40%)	1 (7.1%)	
	3	2 (20%)	13 (93%)	
	4	0 (0%)	0 (0%)	
	5	0 (0%)	0 (0%)	
average number of implanted leads		2.4±0.9	2.9±0.3	0.13

*P* value shows manual traction with retained leads vs excimer laser sheath extraction.

The success rate was 77% and 100% in the patients in whom manual traction succeeded and the patients in whom excimer laser sheath was used to remove a retained lead, respectively (Fig 1). These patients were confirmed using angiography that all targeted leads and all lead material including insulation were removed from the vascular space. Both groups had no patients with intraoperative or perioperative complication associated with lead extraction. There were no significant differences in the background factors, including the lead implantation period, between patients with retained leads (N = 5) and patients who received complete lead extraction using the excimer laser (N = 14) (Table 2). However, comparing patients without retained leads either after manual traction (N = 20) or after excimer laser sheath extraction (N = 14), the lead implantation period was significantly longer in the latter patient group (manual vs. laser, 1532±187 days vs. 2099 ± 219 days; *P* = 0.03). Otherwise, there was no significant difference in other characteristics (Table 3).

**Table 3:** Background in patients without retained leads.

		manual traction (N = 20)	excimer laser sheath extraction(N = 14)	P value
type of device	ICD	2 (10%)	0 (0%)	0.63
	CRTD	14 (70%)	14 (100%)	0.07
	CRTP	3 (15%)	0 (0%)	0.37
	PM	1 (5%)	0 (0%)	0.4
median time from device implantation to OHT (days)		1532±187	2099±219	0.03*
type of defibrillator lead	single coil	0 (0%)	3 (21%)	0.12
	dual coil	14 (70%)	11 (79%)	0.87
number of leads at time of OHT	1	2 (10%)	0	
	2	3 (15%)	1	
	3	14 (70%)	13	
	4	0 (0%)	0	
	5	1(5%)	0	
average number of implanted leads		2.9±0.9	2.9±0.3	0.25

*P* value shows manual traction vs excimer laser sheath extraction.

*P<0.05 vs manual traction.

Regarding comorbidity, 33 patients (39%) experienced symptomatic cerebral infarction and bleeding (22 cases with symptomatic cerebral infarction and 15 cases with symptomatic intracerebral hemorrhage), and the rate of the patients who would require an MRI scan was higher in these patients (Table 4).

**Table 4:** Comorbidities in patients.

		overall (n = 84)
embolic stroke		34 (40%)
	symptomatic	22 (26%)
	asymptomatic	12 (14%)
hemorrhagic stroke		18 (21%)
	symptomatic	15 (18%)
	asymptomatic	3 (3.6%)
fracture		6 (7.1%)
gastrointestinal disease		6 (7.1%)
lung disease		5 (6.0%)
gynecological disease		4 (4.8%)
neurological disease		4 (4.8%)
renal infarction		3 (3.6%)
gastrointestinal bleeding		4 (4.8%)

## Discussion

Given the rapidly growing number of patients with old CIED leads, the management of nonfunctional leads is a rising problem. In particular, the implications of abandoned lead extraction are controversial. According to a cohort study performed in 78 patients who underwent ICD implantation, there was no new clinical complication due to retained ICD leads [5]. On the other hand, there is a report that retained leads cause venous thrombosis and oversensing by new leads [6].

**Fig 3:**
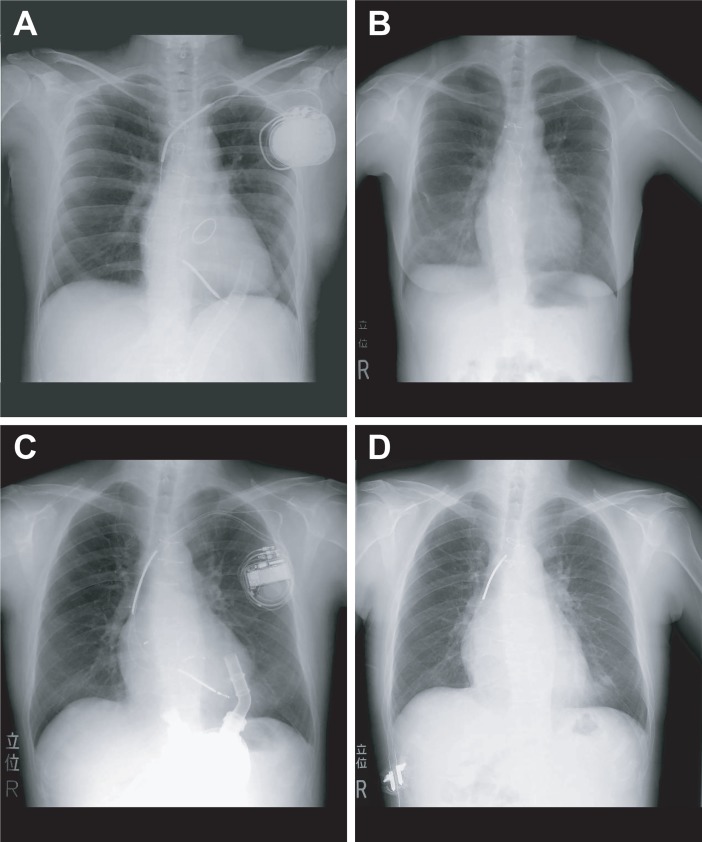
The patients with the retained fragment in superior vena cava after OHT. (A) Chest X-ray of a patient who had an ICD with dual coil shock lead before OHT, (B) Lead fragment from a dual coil defibrillator in the patient shown in (A), as observed in the subclavian vein after OHT. (C) Implantation of a CRTD with dual coil shock lead. (D) Retained distal shock coil in superior vena cava after OHT.

Generally, distal lead fragments are cut off at the vena cava superior during OHT, and retained leads are manually extracted or abandoned. However, some reports show that abandoning a CIED lead following OHT did not cause adverse clinical complications [3, 5]. However, the long-term prognosis associated with retained leads remains unclear. Considering potential future complications including MRI compatibility, device infection, lead migration and lead erosion, some papers insisted that retained leads should be completely removed after OHT [3, 4, 7]. CIED leads strongly adhere to blood vessels, and manual traction of leads is more likely to result in damaged and retained leads; in particular, the proximal coil of a dual coil lead tends to be preferentially abandoned in the subclavian vein, innominate veins, and/or superior vena cava (Fig 3). Although the residual fragments can be removed from the incision of innominate vein, hemostasis is very difficult to achieve during OHT. Moreover, bacteria have been reported to have a tendency to attach to the retained lead site [8]. Regarding damaged leads that are abandoned in patients who receive long-term immunosuppressive treatment after OHT, the risk of infection caused by retained fragments is considered to be high.

Recent reports demonstrated the safe removal of abandoned leads that could not be extracted manually due to their adherence to the venous system using a snare catheter after OHT [2, 3]. However, percutaneous removal of any abandoned lead fragments via a separate operation after OHT must be performed with a surgical backup in case of blood vessel perforation. Moreover, the rate of unsuccessful CIED removal and adverse infections associated with immunosuppressant treatment might be increased in patients with long-term implanted CIEDs. Thus, we attempted to completely remove retained leads using the excimer laser sheath method during OHT to avoid complications due to abandoned lead fragments.

In order to stop bleeding in case of venous damage, our method involved performing the lead extraction using the excimer laser before chest closure. As such, we can perform laser extraction with cardiac surgical backup and provide rapid surgical treatment if vessel perforation occurs. The complete extraction of retained lead using this method during OHT provides the best option in terms of safety precautions and its efficacy.

A predictor for incidence of retained lead fragments with manual traction is the duration from CIED implantation to OHT, which is typically >18 months. Implantation of two or more leads and dual coil leads also trends towards a higher incidence [3]. The waiting period for heart transplantation in Japan is usually longer than 2–3 years due to severe donor shortage [9]. In our study, the mean duration from CIED implantation to OHT is also a long period of 59 months, which is longer than that in the other reports [2, 3, 8]. The mean implantation period in our patients who underwent successful removal of all leads using the excimer laser sheath was approximately 70 months, and the success rate of complete removal of leads was 100% (vs. 53%, 61% and 80.5% in three retrospective studies) without any complications, which indicated relatively favorable results [2, 3, 8]. In addition, the number of implanted leads was higher than that in other studies (2.9 vs. 2) [3, 8]. Retained lead extraction by the excimer laser sheath during OHT is effective and safe regardless of the lead implantation period.

Adverse effects of retained leads on MRI compatibility are also a fundamental problem. There were reports that residual fragments had no influence on MRI scans [10]. However, recently, it has been reported that the temperature of retained lead fragments is elevated during MRI scans, and therefore, MRI scans are not recommended in such cases [11, 12]. In our patients who underwent OHT, patients had comorbidities including symptomatic cerebral infarction (26%), symptomatic cerebral hemorrhage (18%), digestive system disease (12%), and gynecologic disease (5%). MRI scaning may be required for some of the comobidities. Aiming to completely remove retained leads is thought to be necessary in order to conduct MRI scans after OHT.

## Conclusion

Removal of retained leads using the excimer laser sheath during heart transplantation is safe and effective. Retained leads are a contraindication for MRI scans and can complicate the use of MRI scans in future evaluations of patients after OHT. Therefore, complete removal of retained leads is desirable to reduce future potential risks.

## Potential limitations of this study

First, this study is a historical cohort study and is not a prospective study. Second, we analyzed the data of a relatively small sample size from a single institution. Therefore, careful attention should be paid when results are generalized and extrapolated. Thus, larger scale, multicenter surveys will be needed to confirm these results.
